# Hepcidin Is an Antibacterial, Stress-Inducible Peptide of the Biliary System

**DOI:** 10.1371/journal.pone.0016454

**Published:** 2011-01-24

**Authors:** Pavel Strnad, Peggy Schwarz, Maria C. D. Rasenack, Ozlem Kucukoglu, Rayan I. Habib, Dominik Heuberger, Robert Ehehalt, Michael W. Müller, Adolf Stiehl, Guido Adler, Hasan Kulaksiz

**Affiliations:** 1 Department of Internal Medicine I, University Hospital Ulm, Ulm, Germany; 2 Department of Internal Medicine, Division of Gastroenterology, University Hospital Heidelberg, Heidelberg, Germany; 3 Department of General and Visceral Surgery, Medical Center Stuttgart, Stuttgart, Germany; University of South Florida College of Medicine, United States of America

## Abstract

**Background/Aims:**

Hepcidin (gene name *HAMP*), an IL-6-inducible acute phase peptide with antimicrobial properties, is the key negative regulator of iron metabolism. Liver is the primary source of *HAMP* synthesis, but it is also produced by other tissues such as kidney or heart and is found in body fluids such as urine or cerebrospinal fluid. While the role of hepcidin in biliary system is unknown, a recent study demonstrated that conditional gp130-knockout mice display diminished hepcidin levels and increased rate of biliary infections.

**Methods:**

Expression and localization of *HAMP* in biliary system was analyzed by real time RT-PCR, in-situ hybridization, immunostaining and –blotting, while prohepcidin levels in human bile were determined by ELISA.

**Results:**

Hepcidin was detected in mouse/human gallbladder and bile duct epithelia. Biliary *HAMP* is stress-inducible, in that it is increased in biliary cell lines upon IL-6 stimulation and in gallbladder mucosa of patients with acute cholecystitis. Hepcidin is also present in the bile and elevated prohepcidin levels were observed in bile of primary sclerosing cholangitis (PSC) patients with concurrent bacterial cholangitis compared to PSC subjects without bacterial infection (median values 22.3 vs. 8.9; p = 0.03). In PSC-cholangitis subjects, bile prohepcidin levels positively correlated with C-reactive protein and bilirubin levels (r = 0.48 and r = 0.71, respectively). *In vitro*, hepcidin enhanced the antimicrobial capacity of human bile (p<0.05).

**Conclusion:**

Hepcidin is a stress-inducible peptide of the biliary epithelia and a potential marker of biliary stress. In the bile, hepcidin may serve local functions such as protection from bacterial infections.

## Introduction

Iron is an essential trace element, that is indispensable for basic cellular processes but is toxic at higher levels and therefore, its metabolism is tightly regulated [1], [2]. This happens primarily at the level of intestinal iron absorption as well as the cycling of the iron between the different compartments [1]. Hepcidin (encoded by the *HAMP* gene) is the key regulator in this process, since it blocks the absorption of iron from the intestine and the release of iron from macrophages through degradation of the iron exporter ferroportin [1], [3], [4]. Accordingly, *HAMP* knockout animals and humans carrying hepcidin mutations display an excessive parenchymal iron overload while animals over-expressing *HAMP* suffer from iron-deficiency anemia [3]–[5]. In addition to being regulated through iron metabolism, hepcidin is also induced during inflammation through the IL6-gp130-STAT3 axis [2] and is thought to improve the host response to pathogens, both through its direct antimicrobial properties as well as through limiting the availability of iron [2], [6], [7]. Consequently, hemochromatosis patients, who exhibit inappropriately low hepcidin levels, do not only suffer from iron overload, but also from increased rates of bacterial infections [2], [8].

*HAMP* is mainly produced by the hepatocytes as an 84 amino acids pre-propeptide, which is first processed to a 60 amino acid prohepcidin and cleaved to its active form, which is secreted into the bloodstream [3]. In addition to liver, hepcidin is made by multiple other tissues such as kidney (hepcidin is present in thick ascending limb of the cortex and in connecting tubules), pancreatic beta cells, adipose tissue or heart (hepcidin is found at the intercalated disc area) [9]–[12]. It is also detected in different body fluids including urine, bile, ascitic, pleural or cerebrospinal fluid [3], [13], [14], however, the cellular source of the secreted peptide still remains to be determined. Although the role of extrahepatic hepcidin production is far from being understood, it is thought to serve iron regulatory and antimicrobial functions [3], [9]–[14].

To follow up on the recently described presence of hepcidin in the bile [13], we investigated the expression, regulation and function of hepcidin within the biliary system. Several recently reported observations make the biliary system an attractive target for hepcidin: (i) the biliary excretion is a significant pathway for iron elimination [15]; (ii) biliary system is at significant risk of bacterial and fungal infections which are to a large extent counteracted by the antimicrobial properties of the bile [16], [17]; (iii) Gp130-deficiency results in diminished hepcidin levels and increased rate of biliary infections [18].

## Materials and Methods

### Tissue samples

All human tissue specimens were obtained from tissue explants performed at the University of Heidelberg. Non-diseased liver, bile duct and gallbladder samples (n = 15 for each tissue) were taken as a part of Whipple surgery performed for an extrabiliary malignancy, during a hepatic tumor surgery or in the context of a liver transplantation. To determine hepcidin expression during inflammation, gallbladders from patients with acute cholecystitis were examined (n = 10) and the presence of acute inflammation was confirmed by histologic evaluation as well as the macroscopic picture. Control bile specimens (n = 15) were obtained during an inconspicuous endoscopic retrograde cholangiopancreatography (ERCP) performed for elevated cholestasis parameters. The presence of biliary infection was excluded by negative bile bacterial culture and normal C-reactive protein (CRP) levels.

To quantify prohepcidin levels in the bile under stress conditions, bile specimens were obtained from 42 patients with primary sclerosing cholangitis (PSC). In 26 of them, a biliary infection was evidenced by the presence of an Enterobacter-positive bile culture, whereas the remaining 16 patients had no detectable biliary infection.

Animal tissues were obtained from 2–6 month old C57BL/6 mice (n = 6; Charles River, Sulzfeld, Germany) as well as adult 4 week old HFD POC DH guinea pigs (Harlan laboratories, Eystrup, Germany) kept on a standard animal diet containing 185 mg iron/kg (sniff-Spezialdiäten GmbH, Soest, Germany). Animals were euthanized by CO_2_ inhalation and tissues were snap frozen in liquid nitrogen (protein biochemistry) or submerged in RNA*later* Stabilization Reagent (Qiagen) and stored at −20°C (RT-PCR). Two tailed student's t-test was used to compare the expression levels in different mouse tissues.

### Ethics statement

The studies on human materials were approved by the Ethics Committee of the University of Heidelberg and the University of Ulm and informed consent was obtained from each patient participating in the study.

### Prohepcidin competitive ELISA assay

To determine prohepcidin concentrations in the bile, a competitive ELISA assay was performed as previously described [19]. Briefly, bile was spun down at 20000 rpm for 10 min and supernatant was collected. 2 ng of N-terminally biotinylated hepcidin (Peptide Specialty Laboratories GmbH, Heidelberg, Germany) was added to each well and mixed with bile samples or synthetic hepcidin standards. After washing out the unbound peptides, amount of biotinylated hepcidin was determined using streptavidin-peroxidase enzyme (Dako, Hamburg, Germany) and tetramethylbenzidine as a substrate (DRG Instruments GmbH, Marburg, Germany). The measurements were carried out in duplicates. A two-tailed Mann Whitney U test was employed to compare biliary prohepcidin levels in PSC patients with and without cholangitis, while a two-tailed Pearson Product Moment correlation test was used to determine the correlation between prohepcidin levels and CRP or serum bilirubin.

### Cell culture

Human cholangiocarcinoma cell lines RPMI-7451 [20] and Mz-ChA-1 [21] or human hepatoma cell line HepG2 were grown in high glucose Dulbecco's modified Eagle's medium (PAA Laboratories, Cölbe, Germany) supplemented with 10% heat inactivated fetal calf serum as well as 1% penicillin/streptomycin and kept at 37°C and 5% CO_2_. For IL-6 stimulation, a fresh medium with or without IL-6 (final concentration 13 ng/ml, stock 10 µg/ml diluted in phosphate buffered saline, pH 7.4) was added and cells were harvested 4 hours afterwards. To determine the impact of IL-6 on hepcidin expression, a two-tailed Student's t-test was used.

### Qualitative RT-PCR and quantitative real time RT-PCR

To determine hepcidin expression levels, RNA was isolated with the RNeasy Mini-Kit (Qiagen, Valencia, CA, USA) and transcribed into cDNA with the Superscript-III First-strand Synthesis system and Oligo-dT primers (Invitrogen; Carlsbad, CA). Real-time RT-PCR reactions were carried out with SYBR green Master Mix (Applied Biosystems; Foster City, CA). The reactions were performed in duplicate and hepcidin levels were expressed as a ratio to the endogenous loading control (ß-actin). The following primers were used: Human hepcidin (Accession Nr. NM021175) 5′-GAC GGG ACA ACT TGC AGA GC-3′, 5′-GCC TCT GGA ACA TGG GCA-3′; mouse hepcidin (Accession Nr. NM032541.1) 5′-AACAGATACCACACTGGGAA-3′, 5′-CCTATCTCCATCAACAGATG-3′; Human ß-actin (Accession Nr. NM001101) 5′-AGG ATG CAG AAG GAG ATC ACT G-3′, 5′-GGG TGT AAC GCA ACT AAG TCA TAG -3′; mouse ß-actin (Accession Nr. NM007393.3) 5′-CCAAAAGCCACCCCCACTC-3′, 5′-GGGGACAAAAAAAAGGGAGG-3′.

For qualitative RT-PCR, 50 ng of cDNA were PCR-amplified and products were visualized with ethidium bromide on a 1.8% agarose gel [9]. The following human hepcidin primers were used: 5′-CTG CAA CCC CAG GAC AGA G-3′; 5′-GGA ATA AAT AAG GAA GGG AGG GG-3′.

### In-situ hybridization

To visualize the site of hepcidin expression, in-situ hybridization was performed as described previously [22]. Briefly, human hepcidin DNA probes were PCR-amplified from HepG2 cells cDNA using primers with an incorporated T7 recognition sequence: 5′-ctaatacgactcactatagggCAGACACCAGAGCAAGCTCAA-3′ (forward antisense primer for sense probe), 5′-TGGGGCAGCAGGAATAAATAA-3′ (reverse antisense primer for sense probe), 5′-CAGACCACCAGAGCAAGCTCAA-3′ (forward sense primer for antisense probe) and 5′-ctaatacgactcactatagggTGGGGCAGCAGGAATAAATAA-3′ (reverse sense primer for antisense probe; small letters indicate the T7 promotor sequence).

DNA probes were transcribed into RNA and digoxigenin-labelled with a T7 RNA polymerase and DIG RNA-labeling reagents, respectively (Roche Diagnostics, Indianapolis, IN). 200 µg/ml of the resulting antisense or sense probes were hybridized to deparaffinized and dehydrated tissue sections, which were pre-incubated with 1% hydrogen peroxide and pre-digested with a proteinase K (10 µg/ml; 37°C; 30 min). Endogenous biotin was blocked with a biotin-blocking reagent (Dako North America) and the digoxigenin signal was amplified with an anti-digoxigenin antibody and a secondary streptavidin complex (GenPoint kit; Dako). The final signal was developed with diaminobenzidine and a counterstaining with hematoxylin was perfomed to visualize the nuclei.

### Immunohistochemistry

Immunohistochemical staining was performed as described elsewhere [19]. Briefly, paraformaldehyde-fixed (4% for 18 hours), paraffin-embedded 5 µm thin sections of non-diseased gallbladders were incubated with the previously described hepcidin antibodies (24 hours, 4°C) [19], washed twice in PBS, followed by incubation with biotinylated anti-rabbit IgG for 30 minutes (Vector Laboratories, Burlingame, CA). The sections were subsequently exposed to preformed biotin-peroxidase/streptavidin complex for 30 minutes and the antigen-antibody complexes were visualized with 0.7 mM diaminobenzidine hydrochloride/0.002% H_2_O_2_ in 0.05 M Tris HCl (pH 7.6). To increase the signal, 6 mg/ml ammonium-nickel sulphate was added to the diaminobenzidine–H_2_O_2_ solution wherever indicated [23]. To confirm the specificity of immunohistochemical staining, the previously described control stainings were performed [9]. For example, a pre-adsorption with 6.25 µg/ml of the respective peptide for 30 minutes completely abolished the immunohistochemical signal.

### Immunofluorescence

Hepcidin distribution within gallbladder tissue was visualized after incubation of paraformaldehyd-fixed and paraffin-embedded 5 µm thick sections with a novel monoclonal hepcidin antibody mHK(9) (Schwarz et al., in revision) or previously described rabbit polyclonal prohepcidin antibodies [19]. Subsequently, sections were subjected to fluorochrome coupled secondary antibody (Cy2/Cy3 goat anti-mouse/rabbit IgG, Invitrogen, Karlsruhe, Germany). Images were acquired on an Olympus fluorescence microscope (Hamburg, Germany) equipped with a digital camera and SimplePCI software (Hamamatsu Photonics, Herrsching, Germany).

### Bacterial growth assay

To quantify the antimicrobial properties of hepcidin, *E. coli* bacteria (strain DSM 1058) were brought to the exponential growth phase and incubated with recombinant hepcidin (100 µg/ml; Bachem, Weil am Rhein, Germany) or bile (filtered through a 10 kD protein filter; Millipore, Schwalbach/Ts, Germany) obtained during inconspicuous ERCP alone (10 µl bile/100 µl bacterial culture) or with both agents in combination for 1 hour. Serial dilutions of the resulting bacterial cultures were generated and grown on agar plates at 37°C. After 24 hours, the number of colony forming units was counted. For every experiment, a bile sample from a separate individual was used and four independent experiments were performed.

### Protein biochemistry

30 µg of protein extract was supplied with 0.7% β-mercaptoethanol and heat denaturated (95°C, 7 min) in a 4%(w/v)-sodium dodecylsulphate (SDS)-containing buffer [50 mM Tris-HCl, pH 8.45, 1 mM EDTA, 3.24 mM DTT, 12.5% (w/v) glycerol, 0.002% bromophenol blue, 4% SDS]. Lysates were separated on 16.5% tricine-sodium gels and transferred to a polyvinylidene fluoride membranes (Pall, Porthmouth, UK). Prohepcidin signal was detected through two independent, previously described N-terminal prohepcidin antibodies [19] in combination with alkaline phosphatase-coupled secondary antibody and visualized with nitro blue tetrazolium and 5-bromo-4-chloro-3-indoyl phosphate (Sigma).

## Results

### Hepcidin is expressed in the biliary epithelia and found in the bile

**Figure 1:**
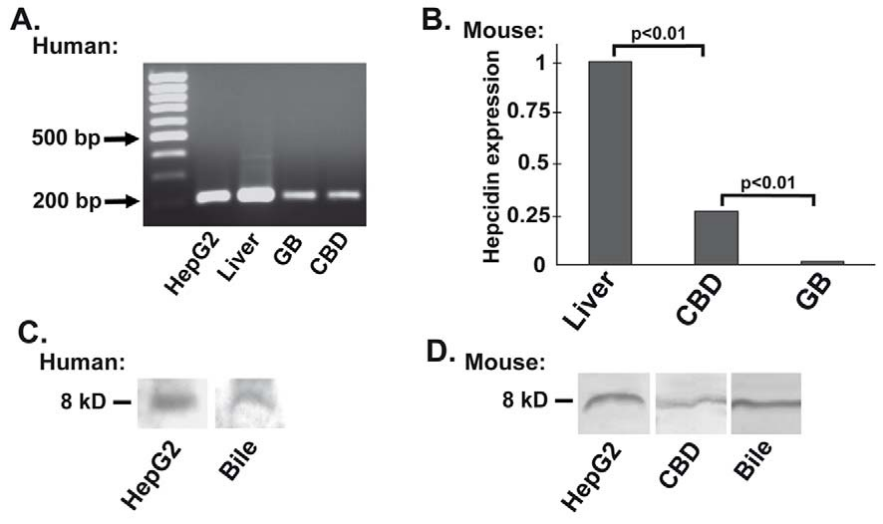
Hepcidin is expressed in the biliary system and found in the bile. (*A*) Qualitative RT-PCR analysis reveals hepcidin mRNA expression in human hepatocellular carcinoma cell line (HepG2) as well as in human liver, gallbladder (GB) and common bile duct (CBD). The size of the amplified fragment was determined using a 100 bp DNA ladder (lane 1). Tissues from at least 15 individuals were examined to confirm the presented expression levels. (*B*) Real time RT-PCR was employed to quantify the mouse hepcidin mRNA levels. Results were compared to β-actin, which was used as an internal control and liver expression level was arbitrarily set as 1. Samples from at least five individual mice were examined. (*C and D*) Western blotting with two independent N-terminal antibodies (*C,D*) confirmed the presence of hepcidin peptide in HepG2 cell extracts (used as a positive control) as well as in human/mouse bile and mouse common bile duct (CBD).

To test, whether hepcidin is present in the biliary system, total RNA was isolated from 15 individual patients. RT-PCR demonstrated hepcidin mRNA presence in both gallbladder and common bile duct (CBD). Liver tissues and HepG2 cell extracts were used as positive control and resulted in strong hepcidin amplification (Fig. 1A), while non-transcribed total mRNA did not reveal any signal thereby confirming the PCR specificity (not shown). Hepcidin 1 mRNA was also present in both mouse gallbladder and CBD with expression levels being liver>CBD>gallbladder (normalized values 1±0.32 vs. 0.26±0.12 vs. 0.02±0.02; Fig. 1B). Hepcidin peptide was observed in both the human and mouse bile as well as mouse CBD and gallbladder as shown by western blotting with three independent antibodies directed against N- and C-terminal part of hepcidin (Fig. 1C,D and not shown). The peptide levels were comparable to levels seen in total cell lysate from HepG2 cells.

**Figure 2:**
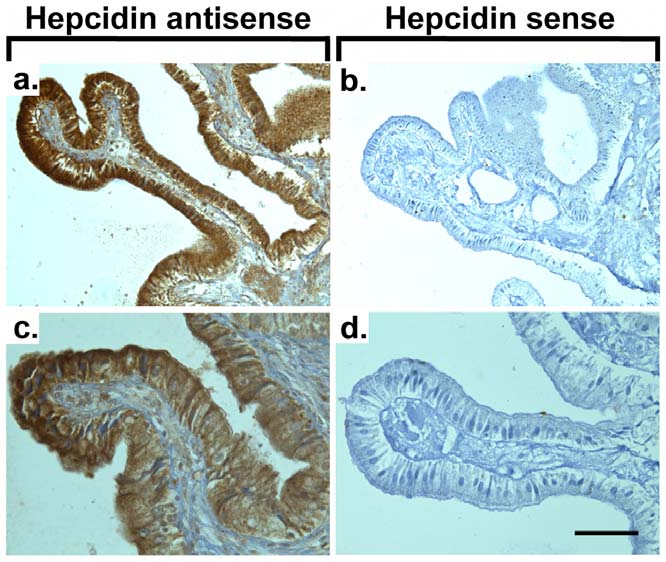
Hepcidin mRNA signal localizes to human gallbladder epithelia. In-situ hybridization of paraffin-embedded sections detected hepcidin mRNA in human gallbladder epithelia (a,b), while no signal was obtained with the sense probes (c,d) thereby confirming the specificity of the staining. To visualize the nuclei, sections were counterstained with hematoxylin. Scale bars 100 µm (a,b); 50 µm (c,d).

**Figure 3:**
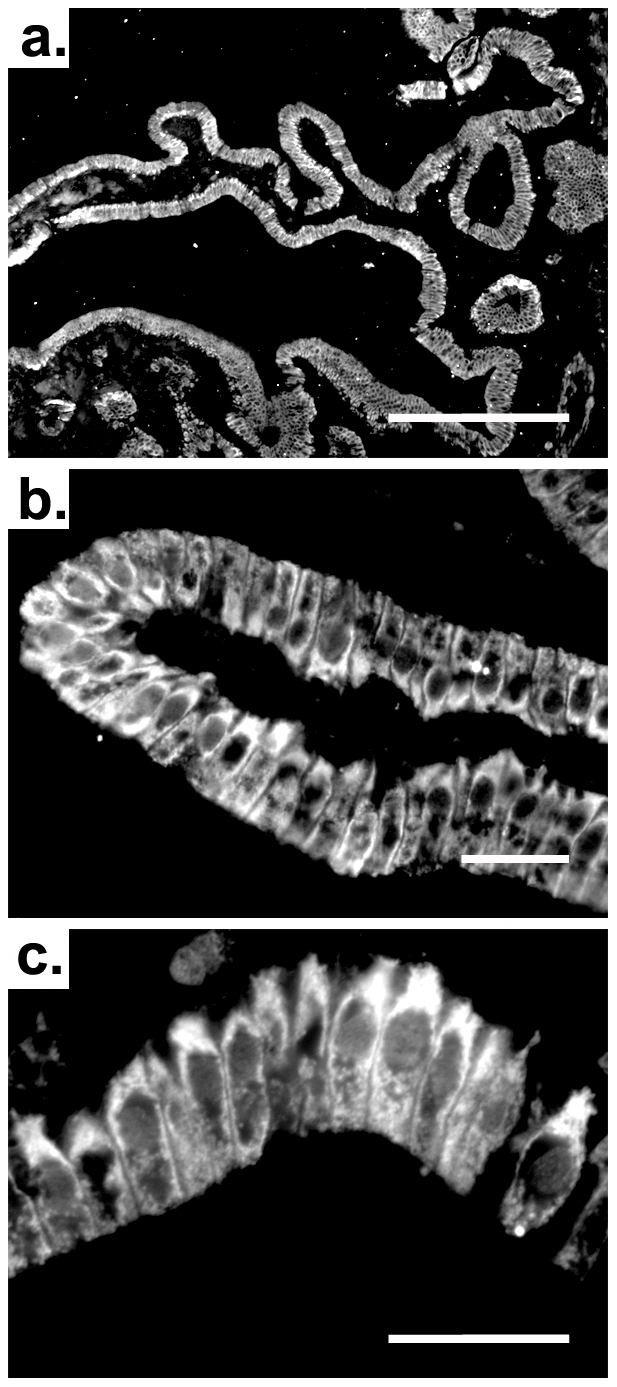
Immunofluorescence staining reveals hepcidin in the cytoplasm of human gallbladder epithelia. To characterize the hepcidin localization within non-diseased human gallbladder, an immunofluorescence staining with a novel monoclonal hepcidin antibody was performed. Please note the cytoplasmic localization of the antigen, which is restricted to the epithelial cells. Scale bars 500 µm (a); 50 µm (b,c).

**Figure 4:**
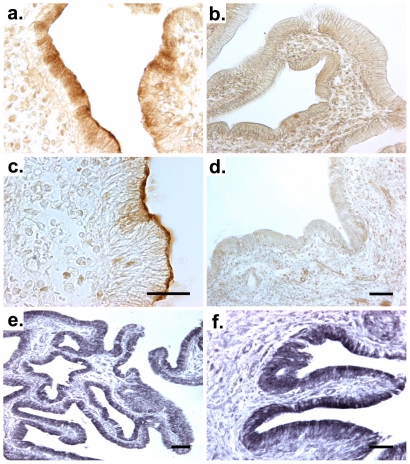
Hepcidin is preferentially found in the apical membrane domain of the gallbladder epithelial cells. Cellular localization of hepcidin in human gallbladder was visualized by immunohistochemical staining of paraffin sections with diaminobenzidine with (e–f) or without (a–d) ammonium-nickel sulphate enhancement. The staining revealed that, in some cells, immunoreactivity for hepcidin is evenly distributed throughout the cytoplasm of epithelial cells (e,f), while in different gallbladder cholangiocytes, the epitope is enriched at the apical domain of gallbladder epithelia (c). Similar results were observed with two different C-terminal (a,c) and one N-terminal prohepcidin antibody (e,f). Of note, the presented staining is specific, since it is completely blocked by pre-incubation of the antibodies with their respective immunogenic peptides (b,d). Scale bars 50 µm (a,b,d); 30 µm (c); 100 µm (e); 50 µm (f).

To determine the site of hepcidin expression, we used in-situ hybridization, which located hepcidin mRNA to gallbladder epithelial cells in humans (Fig. 2a,c), whereas no immunoreactivity was seen in the subepithelial or muscular layers. No signal was obtained with the sense probes (Fig. 2b,d) thereby confirming the specificity of the staining. Immunofluorescence and immunohistochemistry confirmed the data, both showing a strong hepcidin fluorescence in the cytoplasm of non-diseased human gallbladder epithelial cells (Fig. 3,4) as well as in guinea pig gallbladder epithelia (Fig. S1,S2). In some gallbladder cholangiocytes, hepcidin was evenly distributed throughout the cytoplasm whereas it was concentrated to the apical domain in others (Fig. 4). The presented staining was specific since it was completely blocked by pre-incubation of the antibodies with their respective peptide used for the immunization (Fig. 4b,d).

### Hepcidin in the biliary system is stress-inducible

**Figure 5:**
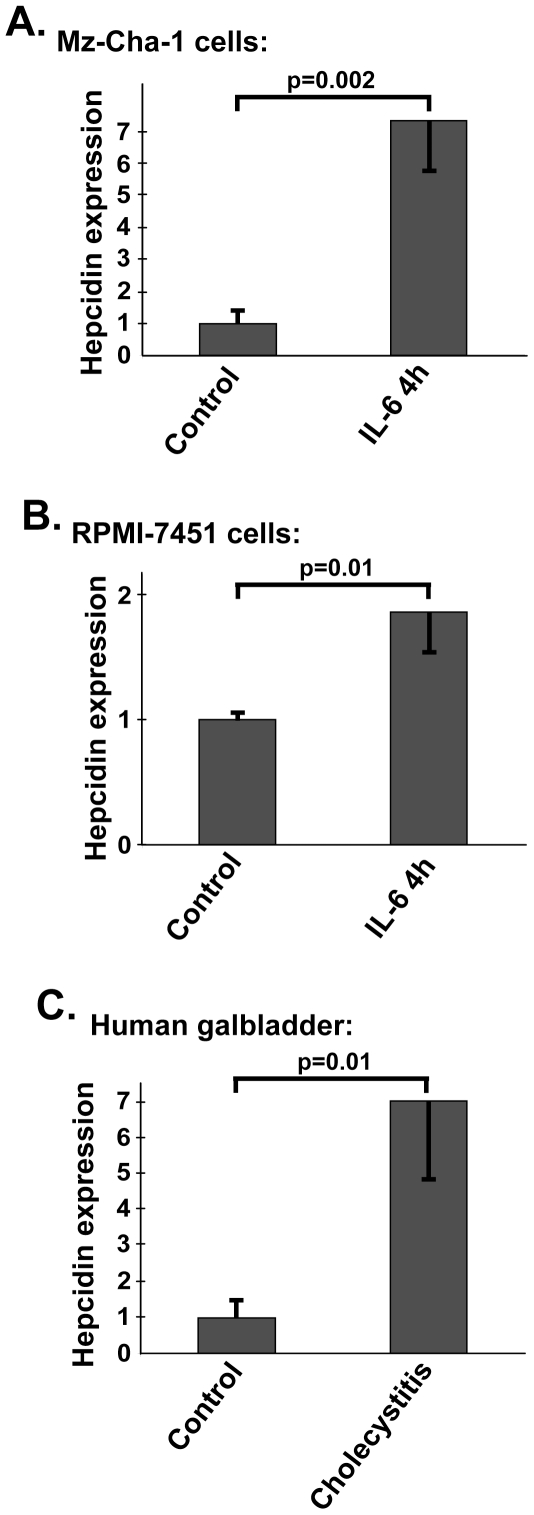
Biliary hepcidin expression is stress-inducible. (*A and B*) Quantitative real-time RT-PCR analyses confirmed elevated hepcidin expression in the biliary cell lines Mz-Cha-1 (A) and RPMI-7451 (B) four hours after IL-6 stimulation (right bar; 13 ng/ml) compared to basal conditions (left bar). The graphs indicate the average values plus standard deviation of four independent experiments. (*C*) Quantitative real time RT-PCR analysis of hepcidin expression in normal gallbladder mucosa (used as a control) and in gallbladder mucosa from patients with acute cholecystitis (average of four samples each). Gallbladder inflammation resulted in ∼7 fold up-regulation of hepcidin mRNA levels. The hepcidin expression was compared to housekeeping gene beta-actin, which was used as an internal control and the expression in non-treated cells/gallbladders was arbitrarily set as 1.

Given that hepcidin is known as an acute phase protein [24], we wondered whether this inflammation-inducible regulation also applies to biliary epithelia. To test that, we stimulated two commonly used cholangiocarcinoma cell lines RPMI-7451 [20] and Mz-ChA-1 [21] with IL-6, an established hepcidin inducer [24]. IL-6 resulted in significantly higher hepcidin mRNA levels in both cell lines although the extent of hepcidin induction differed (80% in RPMI-7451 vs. 700% in Mz-CHA-1 cells, respectively; p = 0.01 and p = 0.002; Fig. 5A,B). To study the hepcidin expression in the whole tissue context, we examined tissue samples from patients with and without acute cholecystitis. Of note, biliary hepcidin expression was elevated ∼7-times in patients with acute cholecystitis (Fig. 5C), in whom the presence of inflammation was confirmed histologically (not shown) when compared to the non-inflamed controls (p = 0.01).

### Hepcidin is a potent antimicrobial component of the bile

**Figure 6:**
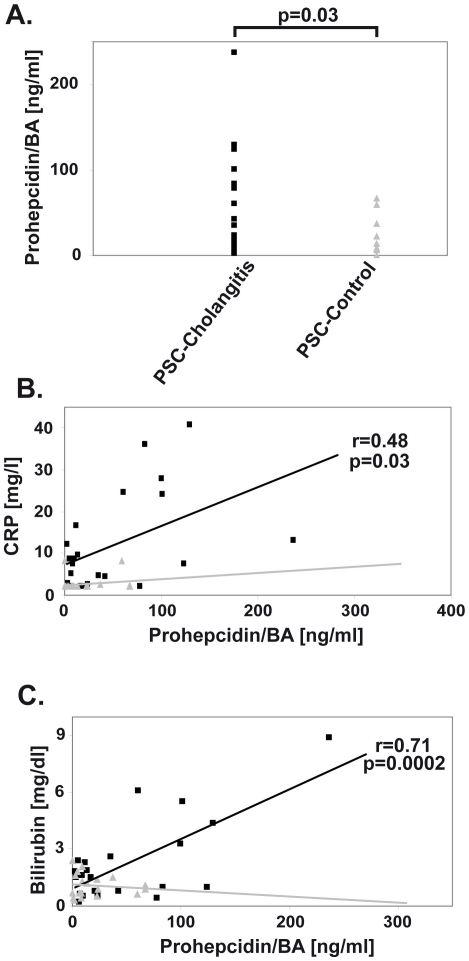
Prohepcidin levels are upregulated in cholangitis patients and correlate with the extent of cholestasis. (*A*) Prohepcidin concentration was measured in bile of PSC patients with acute cholangitis (PSC-cholangitis) or PSC patients with sterile bile (PSC-control) by ELISA. The diagnosis of acute cholangitis was based on the presence of enterobacter in the bile culture together with elevated CRP and/or bilirubin levels. Note that prohepcidin was upregulated in bile of patients with acute cholangitis (p = 0.03). (*B*) Pearson Product Moment correlation revealed significant positive correlation between prohepcidin and C-reactive protein (CRP) levels (r = 0.48, p = 0.03) as well as between prohepcidin and bilirubin levels (r = 0.71, p = 0.0002) in serum of PSC cholangitis patients (black squares and line), while no correlation was observed in PSC controls (grey triangles and line).

To test, whether elevated tissue expression of prohepcidin in the gallbladder epithelia is also translated into increased biliary hepcidin levels, we measured prohepcidin peptide levels in PSC patients with and without a bacterial cholangitis using a previously established prohepcidin ELISA assay [19]. In patients with no apparent biliary infection, prohepcidin levels were below 70 ng/ml (median value 8.9), whereas significantly higher levels were seen in patients with acute cholangitis (up to 240 ng/ml; median value 22.3, p = 0.03; Fig. 6A). Moreover, in PSC patient with cholangitis, prohepcidin levels correlated with CRP (r = 0.48, p = 0.03) and bilirubin serum levels (r = 0.71, p = 0.0002, Fig. 6B,C) thereby suggesting that prohepcidin levels in the bile reflect the extent of biliary infection. Of note, no correlation between prohepcidin, CRP and bilirubin levels was observed in PSC controls, i.e. patients with chronic liver disorder without presence of bacterial infection.

**Figure 7:**
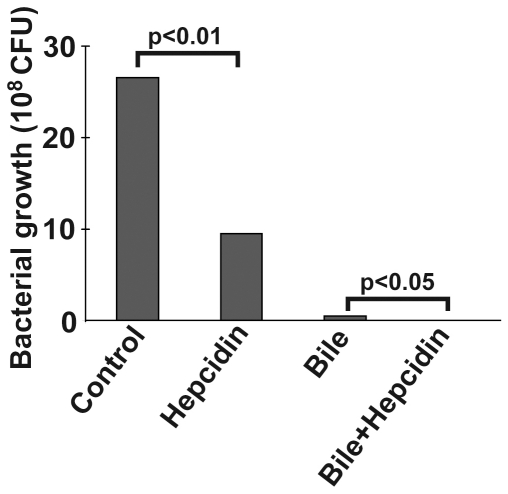
Hepcidin enhanced the antimicrobial capacity of human bile. To quantify the antimicrobial properties of human hepcidin and biliary juice, *E. coli* bacteria were brought to the exponential growth phase and incubated with both agents alone or in combination for 1 hour. Serial dilutions of the resulting bacterial cultures were generated and grown on agar plates for 24 hours at 37°C to allow for formation of colonies. Note that hepcidin exhibited significant antibacterial properties and further increased the antibacterial capacity of human bile juice.

Given that hepcidin is an established antimicrobial peptide [2], [6], we tested whether elevated biliary hepcidin levels seen during cholangitis/cholecystitis may represent a protective antimicrobial response of the organism. To that end, *E. coli* bacteria were brought to the exponential growth phase and were incubated with either hepcidin or bile alone or both agents in combination. As expected, hepcidin alone significantly inhibited *E. coli* growth (p<0.01) and this effect was synergistic with the antibacterial effect of the non-diseased human bile (p<0.05 when comparing the combined treatment with the bile treatment alone; Fig. 7).

## Discussion

Our study establishes hepcidin as a peptide of the biliary epithelia, a finding, which was confirmed in several species (i.e. human, mouse, guinea pig) and using several independent techniques. *HAMP* mRNA expression in common bile duct reaches ∼25% of the expression observed in the liver, which is particularly impressive given the fact that hepcidin expression is restricted to the epithelial/parenchymal cells, which represent the most numerous cell population in the liver, while biliary tract also consists of significant amount of nonepithelial cells such as fibroblasts or smooth muscle cells. Biliary *HAMP* expression is stress-inducible, in that it increased after IL-6 stimulation and during acute cholecystitis. These findings are in line with the IL-6-mediated hepcidin increase in multiple other cell lines as well as the liver [2] and to the previously reported *HAMP* up-regulation during inflammation [11], [12], [25].

Hepcidin is also present in the bile and our data suggest that it may originate from the biliary system rather than from hepatocytes. We demonstrate here that hepcidin is enriched in the apical portion of the cholangiocytes which is compatible with a luminal secretion, while it is found at the basolateral membrane in hepatocytes, which are known to release hepcidin into the bloodstream [5], [19]. Furthermore, biliary prohepcidin levels positively correlate with bilirubin levels in patients with PSC-cholangitis, whereas cholestasis was shown to downregulate *HAMP* expression in the liver [26]. Our data are supported by other reports showing that hepcidin content in body fluids does not mirror the hepcidin serum levels. For example, hepcidin levels in the serum and the urine display different diurnal pattern [27] and the *HAMP* expression in choroid plexus was shown to correlate with hepcidin levels in cerebrospinal fluid during LPS-induced inflammation [14].

The local, stress-inducible production of hepcidin in the biliary system makes it a potential marker of the activity of biliary disorders. This is supported by our data showing that prohepcidin levels in the bile are elevated in patients with PSC-associated cholangitis, in whom they tightly correlate with serum CRP and bilirubin levels, the two important parameters of disease severity. We believe that the diagnostic utility of hepcidin may extend to other body fluids and that local hepcidin levels are more suitable to mirror the neighbouring pathological processes than the serum hepcidin levels, which may be less specific and are affected by multiple factors such as hypoxia, inflammation or iron status [2], [3]. Accordingly, urinary hepcidin levels were recently shown to mirror the activity of renal diseases such as human lupus nephritis or murine nephrotoxic serum nephritis [28], [29].

The presence of hepcidin in body fluids raises the question what might be its function within these compartments. Given the established role of hepcidin as an antimicrobial agent [6] and the fact that body fluids play an important role in defence against bacterial infections, a participation of hepcidin in this process is conceivable. This suggestion is supported by the finding that hepcidin displays >4x higher values in exudative fluids compared to transsudates [13] as well as by our finding correlating the increased hepcidin levels in the bile with the extent of PSC-cholangitis (see above). Since the defence against bacteria is of a particular importance for the bile, which needs to stay sterile despite its direct connection to the bacteria-rich duodenum [17], we tested whether hepcidin plays a role in this process. Here we show that the antibacterial function of hepcidin is additive to the effect of human bile, thereby extending earlier findings describing hepcidin as an antimicrobial peptide [6].

In summary, our study defined the expression and regulation of hepcidin within the biliary system, showed that it represents an attractive marker reflecting the extent of the biliary inflammation (as shown for patients with cholecystitis and PSC-associated bacterial infection) and may play a role in the biliary response to bacterial infections. Further studies are warranted to analyze the potential of hepcidin as a biliary stress marker as well as to more directly evaluate its contribution to the defence against biliary infections.

## Supporting Information

Figure S1 **Immunohistochemistry visualizes hepcidin in the guinea pig gallbladder epithelia.** Immunohistochemical staining with diaminobenzidine detects hepcidin in the gallbladder epithelial cells, while subepithelial layers display no/only minimal signal. These results were observed with a C-terminal (a) and a N-terminal antibody (b). Scale bar 100 µm.
(TIF)

Figure S2 **Immunofluorescence staining localizes hepcidin to the guinea pig gallbladder epithelia.** An indirect immunofluorescence staining reveals strong hepcidin signal in the guinea pig gallgladder epithelia, while no labelling is seen in the subepithelial levels. These results were observed with a N-terminal (a,b) and a C-terminal antibody (c). Scale bars 50 µm (a); 20 µm (b,c).
(TIF)
