# Supplementary figures and images for: Hepcidin Is an Antibacterial, Stress-Inducible Peptide of the Biliary System
Source: PLoS One. 2011 Jan 24;6(1):e16454. doi: 10.1371/journal.pone.0016454 (PMC3025980; doi:10.1371/journal.pone.0016454)

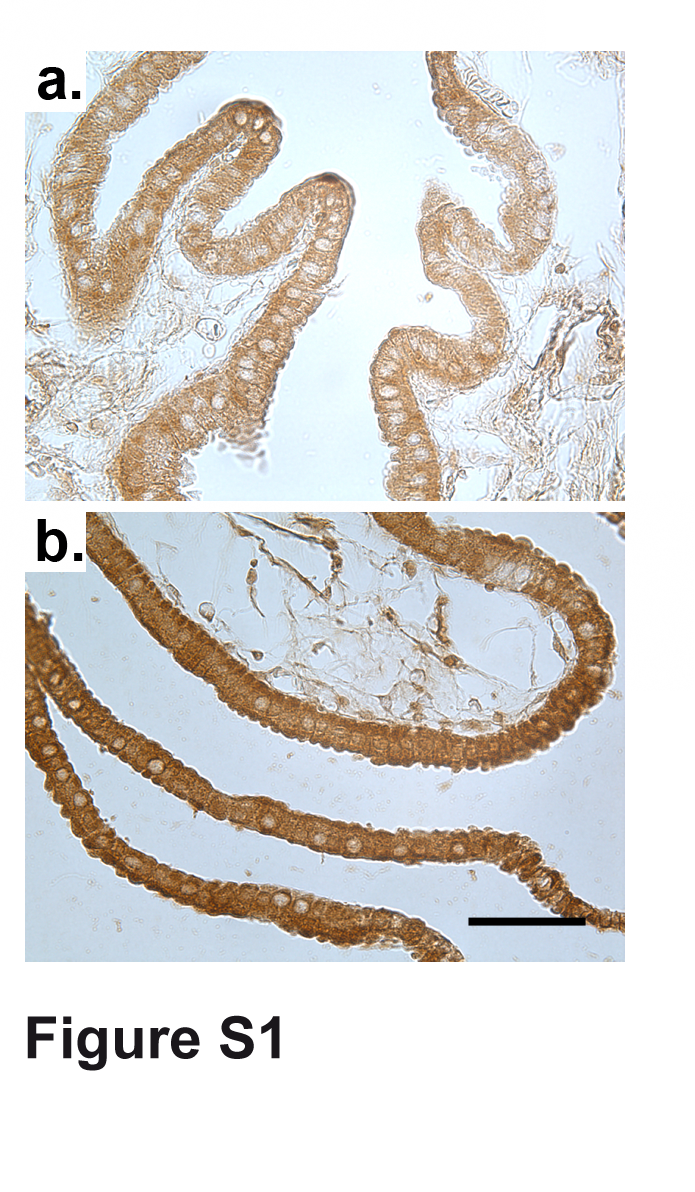

Supplement: Figure S1 — Immunohistochemistry visualizes hepcidin in the guinea pig gallbladder epithelia. Immunohistochemical staining with diaminobenzidine detects hepcidin in the gallbladder epithelial cells, while subepithelial layers display no/only minimal signal. These results were observed with a C-terminal (a) and a N-terminal antibody (b). Scale bar 100 µm. (TIF) [file pone.0016454.s001.tif]

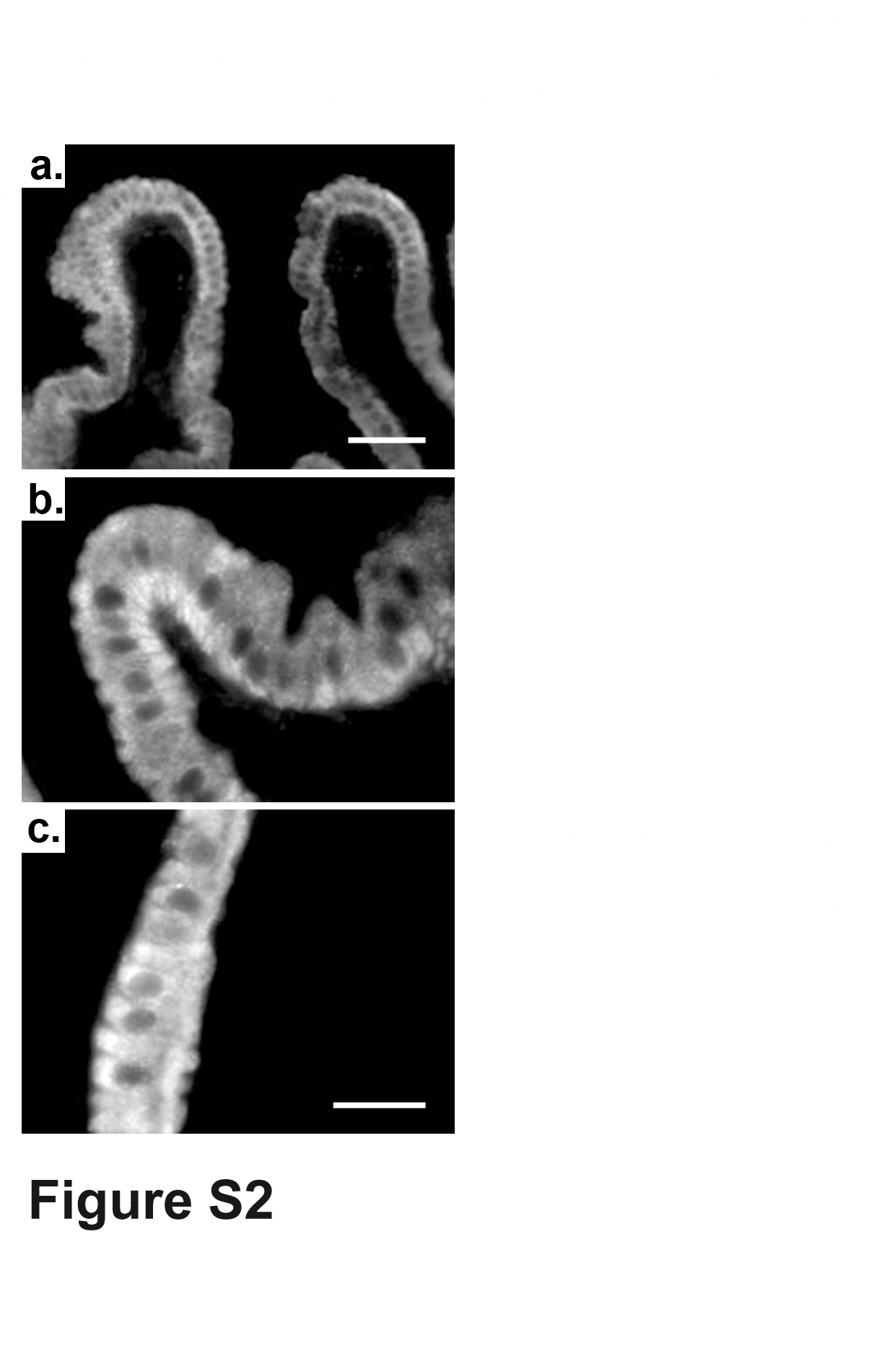

Supplement: Figure S2 — Immunofluorescence staining localizes hepcidin to the guinea pig gallbladder epithelia. An indirect immunofluorescence staining reveals strong hepcidin signal in the guinea pig gallgladder epithelia, while no labelling is seen in the subepithelial levels. These results were observed with a N-terminal (a,b) and a C-terminal antibody (c). Scale bars 50 µm (a); 20 µm (b,c). (TIF) [file pone.0016454.s002.tif]
